# Tissue-Based Approaches to Study Pharmacodynamic Endpoints in Early Phase Oncology Clinical Trials

**DOI:** 10.2174/138945012803530062

**Published:** 2012-11

**Authors:** Joo Ern Ang, Stan Kaye, Udai Banerji

**Affiliations:** The Institute of Cancer Research, Sutton, SM2 5NG, UK and The Royal Marsden NHS Foundation Trust, Sutton, SM2 5PT, UK

**Keywords:** Clinical trials, molecular targeted agents, oncology, pharmacodynamic biomarkers.

## Abstract

Anti-cancer clinical drug development is currently costly and slow with a high attrition rate. There is thus an urgent and unmet need to integrate pharmacodynamic biomarkers into early phase clinical trials in the framework provided by the “pharmacologic audit trail” in order to overcome this challenge. This review discusses the rationale, advantages and disadvantages, as well as the practical considerations of various tissue-based approaches to perform pharmacodynamic studies in early phase oncology clinical trials using case histories of molecular targeting agents such as PI3K, m-TOR, HSP90, HDAC and PARP inhibitors. These approaches include the use of normal “surrogate” tissues such as peripheral blood mononuclear cells, platelet-rich plasma, plucked hair follicles, skin biopsies, plasma-based endocrine assays, proteomics, metabolomics and circulating endothelial cells. In addition, the review discusses the use of neoplastic tissues including tumor biopsies, circulating tumor DNA and tumor cells and metabolomic approaches. The utilization of these tissues and technology platforms to study biomarkers will help accelerate the development of molecularly targeted agents for the treatment of cancer.

## INTRODUCTION

Oncology drug development is exploiting the rapidly evolving advances in our understanding of the biology of cancer by targeting the molecular pathological aberrations which drive progression of individual neoplasms [1]. Notable advances have been made in the technologies to identify and validate anti-cancer targets, in addition to expediting the selection of a potent, selective, pharmacokinetically and pharmacodynamically optimized clinical candidate [2]. Significant improvements have also been made in the molecular characterization of individual tumors. For instance, DNA copy number assessment and gene expression profiling using comparative genomic hybridization and microarray, coupled to mutation analysis using focused resequencing, offers a high level of resolution and reproducibility in aiding understanding of the underlying genetic alterations of specific tumors. Despite these advances the success stories are paradoxically more of an exception rather than the rule. Indeed, the overall clinical development of oncology therapeutics is generally costly, slow and ineffective. 

The median cost of clinical trial participation generally (from Phase I to III) for each patient, as per manufacturers’ estimates, has risen by about 50% to just under £10,000 from 2005 to 2007 [3]; however, the costs of oncology phase I trials are considerably higher. The average time to the United States Food and Drug Administration (FDA) approval from entering Phase I trials is reportedly 7 years, with the time from discovery to reaching the market averaging 10 to 14 years. Although the overall FDA approval rate for new drugs approximates 20%, it was merely 8% for oncology agents between early 1990s and the mid-2000s [4, 5]. 

Overall, the current lack and failure of implementation of a coherent and effective strategy to evaluate novel molecular anti-cancer therapies in patients adds to the challenges of drug development. The improved integration of the use of pharmacodynamic and predictive markers within the framework recommended by the “pharmacological audit trail” is most likely to mitigate risks in drug development. Here, pharmacodynamic markers guide optimal dose and schedule, and predictive markers guide patient selection, in order to improve the design of early phase clinical trials [6, 7].

## EARLY PHASE CLINICAL TRIALS

Phase I oncology clinical trials are conducted to recommend a Phase II dose and schedule by defining toxicity and evaluating the pharmacokinetic / pharmacodynamic profiles. While none of these parameters can be used in isolation, we argue that the incorporation of specific pharmacodynamic endpoints allows drug development to proceed rationally, allowing the examination of pre-specified scientifically-evaluable variables and testing of specific hypotheses [7]. Importantly, the clinical trial designs in current use were formulated in the era of conventional cytotoxic agents and are not customized to develop molecularly targeted agents. It is important to learn from the few success stories, such as those of imatinib and trastuzumab, where their development were guided by the sound understanding of the molecular basis of chronic myeloid leukemia and breast cancer, respectively.

## PHARMACODYNAMIC MARKERS & THE PHARMACOLOGIC AUDIT TRAIL

A widely accepted definition of biomarkers is stated by the United States National Institute of Health Biomarkers Definitions Working Group as “a characteristic that is objective measured and evaluated as an indicator of normal biologic processes, pathologic processes, or pharmacologic response to a therapeutic intervention” [8]. In the context of oncology drug development, the National Cancer Institute in the United States has further classified these into various subtypes, including biological progression, risk/prognostic, predictive & pharmacodynamic biomarkers [9]. Regardless of the subtype, biomarker strategies should be reproducible, minimally invasive with negligible risk when sampled, amenable to repeated sampling and yield sufficient material for analytical studies. It is also vital to emphasize that the biomarker in development needs to be fit-for-purpose, having been analytically and scientifically validated. We shall focus our discussion on the use of pharmacodynamic markers in early phase clinical trials in this review. 

Pharmacodynamic biomarkers characterize the molecular and functional effects produced by an intervention that may or may not correlate with biological and clinical effects, and include molecular, cellular, histopathological, and imaging parameters. These biological effects usually reflect the altered activities or expression of molecular targets in response to a mechanism-based therapy. For example, the demonstration of decreased phosphorylation of a protein substrate immediately downstream from a target kinase is considered a “proximal” pharmacodynamic effect. In addition, studies examining biological effects, such as markers of proliferation and apoptosis, are examples of “distal” effects. The objectives of pharmacodynamic studies include the provision of evidence of mechanism of action of an intervention or drug to guide the selection of the optimal dose and schedule of a drug in conjunction with factors such as pharmacokinetics and drug toxicity [10].

First published in 2002, the pharmacological audit trail provides a conceptual and practical framework to monitor, link and integrate all the key stages in the drug development process through a hierarchy of sequentially connected questions [7]. These include the depiction of target status, effect of body on drug (absorption, distribution, metabolism and elimination pharmacokinetics) and drug on body (including target modulation and changes in downstream biochemical pathway) to subsequent therapeutic and toxicological effects of the drug. The key questions detailed below are relevant to pharmacodynamic biomarkers:

- Is the molecular target expressed or mutated and what is the activity of the pathway?
- Is the drug achieving sufficient concentrations in plasma, blood and tumor tissue?
- Is there activity on the desired molecular target?
- Is there modulation of the biochemical pathway in which the target functions?
- Is there achievement of the desired biological effect?
- Do the above effects translate into a relevant clinical response?

## TYPES OF TISSUES USED IN PHARMACODYNAMIC STUDIES

Tissues and biofluids for biomarker studies can be obtained from several sources. Whilst tumor tissue can be assessed by biopsy, further insights into changes in the tumor can be sought by studying circulating tumor cells and nucleic acids, and intra-tumoral metabolic changes. Normal “surrogate” tissues can be obtained from peripheral blood mononuclear cells (PBMCs), platelet-rich plasma, plucked hair follicles, skin biopsies, oral buccal swaps, metabolomic and endocrine changes in plasma and platelet-rich plasma. Examples of novel targets that are discussed in this review include poly(ADP-Ribose) polymerase (PARP), CYP17, PI3-Akt-mTOR, heat shock protein (HSP)-90 and histone deacetylase (HDAC).

## TUMOR VERSUS NORMAL “SURROGATE” TISSUE

Tumor biopsies are considered to be the gold standard for assessing molecular changes in tissue to guide oncology drug development. The main advantages of this approach include (i) the high likelihood of tumor specimens being molecularly and therapeutically relevant (as opposed to normal “surrogate” tissues); for example, mutations of epidermal growth factor receptor (EGFR) are present in the DNA of tumor tissue of non small cell lung cancer (NSCLC) but not in DNA from normal lung tissue in the same individuals [11], and (ii) the fact that most of the experience in the use of current standard pharmacodynamic assays and methodologies was gained from the use of tumor tissues. However, inherent disadvantages of tumor biopsies include (a) concerns regarding their invasiveness and the implicated procedural risks, although there is little evidence that such biopsies pose an increased risk (indeed, the majority of patients consider the associated potential risks to be acceptable) [12-16], (b) limited scope for a repeated sampling strategy, and (c) heterogeneity within the primary tumor and between tumor sites [17, 18]. These weaknesses partly account for the increasing uptake of the use of normal “surrogate” tissues into clinical trials.

The use of normal “surrogate” tissues for biomarker studies in Phase I trials offer distinct advantages, including (a) low incurred procedural risks, (b) amenability to repeated sampling, thus allowing the temporal characterization of pharmacodynamic effects, (c) the possibility to predict target modulation in tumor tissue based on pharmacokinetic-pharmacodynamic modeling and extrapolation from animal models (an example being the use of PBMCs in the development of everolimus, discussed below [19, 20]), and (d) facilitation of phamacogenomic studies using germ line DNA. The limitations of this approach include (i) the absence of the relevant oncogenic target and associated molecular pathology, such as acquired somatic oncogenic mutations and associated oncogene addiction or a synthetic lethal effect, (ii) difference in drug concentrations between the “surrogate” and the tumor due to differences in tissue architecture and haemodynamics, and (iii) inability to always link changes in these markers to clinical responses.

In the following section, we shall focus our discussion on the benefits and challenges based on our experience gained in the development and use of pharmacodynamic markers in Phase I oncology clinical trials of a wide range of novel molecular targeting agents (refer to Figs. (**1**) and (**2**)).

## NORMAL “SURROGATE” TISSUES

### a) Peripheral Blood Mononuclear Cells (PBMCs)

PBMCs comprise cells of the innate and adaptive immune systems and are commonly extracted from whole blood using ficoll, a hydrophilic polysaccharide that separates layers of blood, with PBMCs forming a buffy coat under a layer of plasma [21, 22]. They can also be extracted from whole blood using a hypotonic lysis which will preferentially lyse red blood cells [23]. Examples of the utility of PBMCs in Phase I clinical trials of novel molecular agents discussed in this review include everolimus (m-TOR inhibitor), 17-allyamino-17-demethoxygeldanamycin (17-AAG) (HSP90 inhibitor), LAQ824 (HDAC inhibitor) and iniparib (PARP inhibitor). 

#### Everolimus

Pharmacodynamic studies involving the m-TOR inhibitor, temsirolimus, were performed relatively late in its clinical development and this was thought to have contributed, at least in part, to uncertainties regarding its optimal dosage and schedule. This led to the more concerted use of pharmacodynamic markers in the subsequent development of another rapamycin analogue, everolimus [20]. In a two-stage Phase I dose-finding study of everolimus, a dose-response relationship between oral administration of everolimus and inhibition of p70S6K1 in PBMCs (already validated in preclinical models) indicated sustained target inhibition over 7 days at doses of ≥20mg/wk. Coupled to data derived from a syngeneic pancreatic rat tumor xenograft model [24], further pharmacokinetic-pharmacodynamic modeling (on a direct-link model, corrected for human pharmacokinetics) was subsequently used to predict intratumoral target inhibition in order to guide weekly and daily dosing [19, 20]. Important to note is the observation that although p70S6K1 inhibition in PBMCs is an accurate biomarker of m-TOR inhibition, it has a limited ability to predict clinical activity [20].

#### 17-AAG

The use of PBMCs in the development of a first-in-class hsp90 inhibitor, 17-AAG, provide further insights into how the use of pharmacodynamic markers could impact on clinical trial decision-making. Gene expression profiling using cDNA microarrays in human colorectal cell lines led to the determination of a molecular signature of drug activity [25]. The correlation between pharmacodynamic changes in PBMCs and in tumor tissue was established in *in vivo* models [21]. Following this, in the pharmacokinetic-pharmacodynamic driven Phase I study of 17-AAG performed at our institution, tumor biopsies were performed only after the satisfactory demonstration of plasma concentrations above those required for activity in human tumor xenograft models and evidence of pharmacodynamic modulation in PBMCs [21, 26]. 

#### LAQ824

In a Phase I trial studying the intravenous infusion of the histone deacetylase inhibitor, LAQ824, Western blot assays of PBMC lysates were performed [27]. These revealed rapid, consistent and dose-dependent histone hyperacetylation from 24mg/m^2^ with a comparable degree of target modulation comparing post- with pre-treatment tumor biopsies. Although histone hyperacetylation has been widely used as a mechanistic marker of HDAC inhibition, this does not seem to correlate with clinical outcome. Significantly, the observation of significant inhibition of HSP90 chaperone function (with depletion of CRAF and increased expression of HSP72) in more than a third of patients with assessable PBMC effects in this study, adds an additional dimension to our understanding of the mechanism of action of HDAC inhibitors. 

#### Iniparib

The use of target inhibition in PBMCs as a pharmacodynamic endpoint has important limitations, as borne through the clinical development of iniparib (BSI-201). This compound was initially developed on the premise that it achieved its anti-neoplastic effect by covalently binding and inhibiting PARP1. Hence, inhibition of PARP in PBMCs was used as a pharmacodynamic endpoint to confirm target modulation and guide dose selection in early phase clinical trials [28, 29]. However, initial exciting Phase I and II efficacy data suggesting iniparib in combination with chemotherapy improved outcome in patients with advanced triple negative breast cancer were not confirmed in a Phase III randomized controlled trial [28, 30, 31]. Other studies now suggest that the major therapeutic mechanism of action of iniparib is not mediated by PARP 1 or 2 inhibition, in contrast to other competitive inhibitors at the NAD+ binding site of PARP, including olaparib and veliparib [32]. It is also noteworthy that there are no published data to-date that demonstrate the achievement of PARP inhibition in tumoral tissue from patients on iniparib.

### b) Plucked Human Hair Follicles

The feasibility of detecting and quantifying cell cycle and DNA repair related-factors, including Ki67, pRb, p27 and phosphorylated p27, pRb and histone H3 in plucked hair follicles, was previously demonstrated [33]. Their use in the preclinical (PX-866) and clinical (olaparib) contexts are discussed.

#### PX-866

Using PX-866, a wortmannin derivative with potent inhibitory effect on PI3K and efficacy in a range of human tumor xenografts [34], phosphorylation of AKT was shown by immunohistochemistry in the follicles of plucked human hair to be inhibited in culture. In fact, the degree of inhibition was greater in the hair follicles than the corresponding effects in human HT29 colon and A549 non-small-cell lung tumor xenografts [35]. 

#### Olaparib

A reported Phase I study of the PARP inhibitor, olaparib, examined the formation of γH2AX foci in plucked hair follicles pre and post-treatment [36]. The induction of γH2AX foci six hours post-treatment indicated PARP inhibition (as measured by PAR formation using an ELISA-based electrochemiluminescence assay in PBMCs and tumor specimens) was rapidly associated with induction of collapsed DNA replication forks and DNA-double strand breaks, in keeping with preclinical models. Additionally, the induction of γH2AX foci was sustained at all later time points with no significant increase in foci induction at doses above 100 mg bd. This provided confidence in subsequent design of a Phase Ib trial involving the combination of olaparib with other chemotherapeutic agents, whereby the starting dose of the novel agent was derived from the Phase I pharmacodynamic data, utilizing the lower limit of the range at which significant target modulation occurred. 

### c) Skin Biopsies

Two examples of the use of skin biopsies are highlighted here to illustrate key principles in the development of EGFR tyrosine kinase and m-TOR inhibitors. They demonstrate both the benefits and limitations of data obtained from this source.

#### EGFR Tyrosine Kinase Inhibitors

The skin was deemed an optimal surrogate tissue for the assessment of therapies targeting the EGFR pathway, since it is easily accessible and has high levels of basal EGFR expression. In addition, the expression of EGFR-inducible molecules such as phosphor-MAPK, p27^KIP1^ and STAT3 have been previously studied in skin biopsies [37]. Thus, skin biopsies were used extensively in the early clinical trials involving EGFR tyrosine kinase inhibitors including gefitinib and erlotinib [38, 39]. In NSCLC, studies of skin biopsies and tumor samples provided initial proof of target modulation. However, pharmacodynamic target modulation may not necessarily translate into clinical efficacy in an unselected patient population. In the case of gefitinib, despite initial high expectations from Phase I and II studies, Phase III clinical data showed that single-agent gefitinib was no better than placebo in the Iressa Survival Evaluation in Lung Cancer study, and gefitinib in addition to chemotherapy demonstrated no improvements in survival, time to progression, or response rate over chemotherapy [40-42]. The subsequent success of EGFR targeted therapies in recent years has been attributed to the utilization of patient selection strategies based on the study of the mutation or amplification status in tumor tissue [43, 44]. 

#### m-TOR Inhibitor

Studies investigating different weekly and daily oral dosing schedules of everolimus made use of skin and tumor biopsies [45]. Immunohistochemistry revealed dose and schedule-dependent inhibition of S6 and 4E-BP1 phosphorylation and decrease in Ki67 levels, with similar effects observed in skin and tumor. The recommended Phase II dose of everolimus at 10 mg/day was based on these pharmacodynamic data, coupled to toxicity considerations. 

### d) Platelet-Rich Plasma

An ELISA-based Meso Scale Discovery electrochemiluminescence pharmacodynamic assay was first validated in healthy volunteers with particular emphasis on ascertaining their sensitivity, specificity, reproducibility and “fitness for purpose” for a Phase I clinical trial [46]. This was subsequently applied to an ongoing clinical trial of a novel, selective and potent class I PI3K inhibitor, GDC-0941. Whilst all drug-related toxicities were NCI common toxicity criteria grade 1 and preliminary pharmacokinetic data suggest dose-proportional increases in fasting mean maximal plasma concentration and area under the curve, preliminary pharmacodynamic data show decreased levels of phosphorylated-AKT in platelet rich plasma correlated with GDC-0941 plasma concentrations [47].

### e) Endocrine Changes

Abiraterone acetate is a novel, potent, selective and irreversible inhibitor of CYP17, and the use of endocrine assays in pharmacodynamic studies was crucial in its development [48]. A compensatory rise in luteinizing hormone levels despite initial suppression of serum testosterone in the first-in-human study pointed to the need for concomitant castration [49]. When abiraterone acetate was subsequently administered on a continuous schedule to patients with castration-resistant prostate cancer, circulating testosterone, oetradiol and downstream C-21 androgenic steroids were suppressed to levels below the lower limit of detection by conventional assays on treatment. As no treatment-related grade 3 or 4 toxicities were encountered and clinical responses were reported at all dose levels tested, the recommended phase II dose of 1000 mg was selected on the basis of the observation that the rise of upstream steroids (including androstenonedione and DHEA) reached a plateau at doses above 750 mg [50]. This example reinforces our belief that the effective doses and schedules of targeted agents may not be primarily determined by considerations of toxicity.

### f) Circulating Endothelial Cells (CECs)

CEC subsets are essential players in angiogenesis, with circulating endothelial progenitor cells (CEPCs) considered to be bone marrow derived embryonic angioblasts that can differentiate into mature CECs [51-53]. Clinical studies with angiogenesis inhibitors in cancer patients with a range of tumor types suggest that CEC kinetics may be prognostic and may serve as biomarkers of drug efficacy [54]. The evaluation of CECs in a Phase I clinical trial of a C-Met inhibitor has shown that the total CEC counts fall significantly after treatment and led to the introduction of dynamic contrast enhanced-MR imaging to investigate the likely anti-angiogenic properties of the novel agent [55].

A range of immunophenotypically and functionally distinct subsets of CECs and CEPCs have been described [56]. They may play important and different roles in neovascularisation and thus hold promise as novel pharmacodynamic biomarkers for agents that interfere with this process. Moreover, further qualitative molecular characterization of the expression of key CEC targets by immunofluorescence is also now feasible. Collectively, the evidence indicates that the analysis of CEC and CEPC subset kinetics is feasible and holds promise as another pharmacodynamic tool to help examine angiogenesis inhibition.

### g) Plasma Proteomics

Proteomic studies in human cancer have generated innumerable datasets of potential diagnostic, prognostic, and therapeutic significance [57]. Central to most of these studies are two-dimensional polyacrylamide gel electrophoresis (2D-PAGE) and mass spectrometry (MS). Specifically, surface-enhanced laser desorption/ionization time-of-flight (SELDI-TOF)-MS remains the current workhorse for serum or plasma analysis, with other methods including isotope-coded affinity tag technology, reverse-phase protein arrays and antibody microarrays emerging as alternative proteomic approaches. Admittedly, technical challenges are significant: standardized sample collection and preparation, and instrumentation protocols are frequently absent, leading to limited analytical reproducibility. Lack of common public data sets and effective computational and bioinformatic strategies to manage the complex, multi-dimensional data generated by proteomic experiments are but a few of the other obstacles. Notwithstanding these challenges, there is little doubt that the proteomic approach has the potential to identify novel biomarkers in cancer. 

An example of a serum-based study with one of the largest series of individuals published to-date was conducted using matrix-assisted laser desorption/ionization-MS in patients with lung cancer [58]. An algorithm was developed to predict treatment outcomes on a training set of 139 patients, which was then tested on two independent validation cohorts of 67 and 96 patients treated with inhibitors of EGFR tyrosine-kinase activity and on three control cohorts of patients who were not treated with these drugs. For both validation cohorts, the classifier identified patients who showed improved outcomes after drug treatment. Median survival of patients in the predicted favorable survival and poor survival groups was 207 days versus 92 days, respectively, for one validation group, and 306 days versus 107 days for the other. This provided proof of feasibility of a serum-based approach to proteomics which could be extrapolated for use in pharmacodynamic studies.

### h) Biofluid Metabolomics

Metabolomics is an analytical tool used to detect and follow changes of metabolites in biofluids or tissues; metabolic profiles captures endogenous and exogenous influences on a living organism and have been argued to be closer to its functional phenotype compared to changes in DNA, RNA and proteins [59]. Based on pattern recognition and other related multivariate statistical approaches, metabolic profiling (fingerprinting and footprinting) broadly encompasses targeted and untargeted analyses. Recent technological advances in NMR spectroscopy and mass spectroscopy (MS), the two most widely utilized methods in the assessment of metabolites, have improved the sensitivity and spectral resolution of these assays such that the progress in this field has been accelerated over the past few years.

In the context of circulating biofluids, the use of ^1^H-NMR spectroscopy had been previously reported to discriminate between the serum of healthy women and that of women with a diagnosis of ovarian cancer [60]. However, with these profiling methodologies, the biological significance of the implicated metabolites is often unclear. An exception to this is provided by a report of the use of high throughput liquid and gas chromatography-based MS of clinical samples related to prostate cancer, which revealed distinct metabolomic profiles differentiating benign, locally advanced and metastatic prostate cancer [61]. In addition, sarcosine, an N-methyl derivative of the amino acid glycine, was identified as a differential metabolite that was significantly elevated during prostate cancer progression to metastasis and can be detected non-invasively in urine. The biological role of sarcosine was additionally validated using *in vitro* models. The collective evidence therefore points to sarcosine being a potentially important metabolic intermediary of prostate cancer cell invasion. Last but not least, metabolomics could also be used as a biomarker of toxicity profiles with particular patterns of metabolic derangements being associated with specific organ dysfunction [62]; current data associated with this approach are preliminary and await further validation.

## MALIGNANT TISSUES

### a) Tumor Biopsy

There are numerous examples of the use of tumor tissues in pharmacodynamic changes in phase I studies studies. An example is the development of the first-in-class HSP90 inhibitor, 17-AAG, in our institution as an illustration of the utility of tumor biopsies. Following evidence of target inhibition, as evidenced by pharmacodynamic modulation in PBMCs, pre- and 24 hr post-treatment tumor biopsies were taken [26]. Western blotting revealed depletion of CRAF and CDK4 with induction of HSP70 in most patients, and immunohistochemistry was used to show that the increased expression of HSP70 occurred in viable tumor cells within melanoma biopsies. Following the observation of activity in malignant melanoma, mutant BRAF has additionally been shown to be more sensitive to depletion by 17-AAG than its wild-type counterpart [63, 64]. Important to also point out is the fact that the conventionally-defined maximal tolerated dose was not reached and the Phase II dose recommendation was made primarily based on pharmacokinetic / pharmacodynamic considerations.

It is essential to note that there could be marked discrepancies in the pharmacodynamic readouts between tumor and normal “surrogate” tissues, as exemplified in the study of erlotinib in breast cancer. Although skin biopsies revealed post-treatment modulation of molecular markers including p-MAPK, p27 and Ki67, tumor biopsies merely demonstrated inhibition of MAPK phosphorylation with no impact on other biomarkers, such as p27 and Ki67 [38]. This highlights the underlying molecular differences between these disparate tissues and data derived from studies in normal “surrogate” tissues should be interpreted in this context. 

Studies using tumor biopsies can also guide the elucidation of potential mechanisms of resistance. In the Phase I development of everolimus, the level of phosphorylated AKT was found to be increased in tumors after treatment. This was later found to be due to the presence of a feedback loop between m-TOR and insulin receptor substrate-1 (IRS-1) whereby m-TOR inhibition induces IRS-1 expression and abrogates feedback inhibition of the pathway, resulting in AKT activation [65]. Thus, this feedback mechanism may limit the efficacy of m-TOR inhibitors. The combination of m-TOR and PI3K inhibitors is therefore hypothesized to confer greater efficacy in malignancies that are addicted to this pathway and current trials are addressing the feasibility of this approach. 

### b) Circulating Tumor Cells (CTCs)

Recent studies in breast, colon and prostate cancers all point to the clinical significance of circulating tumor cells (CTCs) where numbers at baseline and post-treatment changes are strong predictors of outcome [66-68]. Currently, several criteria for defining and methods for detecting CTCs are being used. For instance, the current FDA-approved system is based on immunomagnetic bead enrichment and immunofluoresence staining, whilst the CTC chip is based on a microfluidic platform [69]. Yet other platforms make use of filtration systems that may be coupled to antibody enumeration [70]. The interpretation of data across different studies using different platforms is fraught with difficulties. Direct comparative/cross validation and prospective studies with standardized definitions of CTCs are urgently needed. The use of CTCs as pharmacodynamic markers need not be confined to the analysis of their numbers; indeed, published work has demonstrated the feasibility and utility of performing fluorescence in-situ hybridization and break-apart assays to annotate specific genetic alterations [71]. Work is ongoing to more accurately molecularly characterize these cells. 

### c) Circulating Nucleic Acids

It has been shown for a number of years that circulating tumor-derived mutant DNA (ctDNA) is present in the cell-free fraction of the blood of individuals with cancer but the reliable detection of ctDNA is extremely challenging especially as it represents only a very small proportion of the total circulating DNA [72]. A recently reported technique named BEAMing (beads, emulsion, amplification and magnetics) was developed in patients with colorectal cancer and early results indicate that this novel methodology can provide an exquisitely sensitive and specific framework for the detection and quantification of ctDNA [73]. However, the disadvantages with this approach are that a marker specific for each individual must be developed and that the designing and testing of mutation-specific probes is very time-consuming. Nonetheless, this represents a significant advancement and validation in larger clinical series with optimization of the methodology is required.

### d) Tumor Metabolomics

The neoplastic metabolome is beginning to be characterized. For instance, using standard metabolomic methods, tumors generally display elevated levels of phospholipids, increased glycolytic capacity with increased utilization of glucose carbons to drive synthetic processes and high glutaminolytic function. Additionally, the use of metabolomic MR spectroscopy imaging as a diagnostic tool has been validated with the notable successes of choline and citrate in breast and prostate cancer, respectively [74-76]. There are also extensive data on the ability of this technology to accurately and non-invasively discriminate between the various types of intra-cranial tumors [77, 78].

As a pharmacodynamic marker with novel therapeutics, several proof-of-concept experiments have been reported so far. For instance, NMR metabolomics have revealed specific metabolic changes in cell lines with the use of imatinib inducing reduced glucose uptake by inhibition of glycolysis, increased mitochondrial metabolism with cell differentiation and reduction in phosphocholine with inhibition of cell proliferation [15]. High resolution MAS NMR have shown that lengthening of fatty acid –CH_2_ chains was associated with apoptosis in cervical cancer, in keeping with results of *in vitro* studies of acute lymphoblastic leukaemia treated with doxorubicin [79]. Studies involving ^31^P-NMR have also been published demonstrating a reproducible and robust metabolic signature of altered phospholipid metabolism with the application of 17-AAG, a potent HSP90 inhibitor, to colon cancer-bearing xenograft models [80].

## CONCLUSIONS

The era of molecular targeting agents is characterized by the widening of the therapeutic indices of anti-cancer agents. Pharmacodynamic studies using multiple tissue types are vital for yielding proof of concept of target modulation, optimizing dose scheduling and providing insights into possible novel mechanisms of action and resistance to these agents. Although tumor tissues are the gold standard for conducting these studies, they have significant limitations and normal “surrogate” tissues play important and complementary roles which are discussed in this review. Looking ahead, the armamentarium of new technology and “omics” platforms will undoubtedly play crucial roles in driving the innovation, development and refinement of molecularly targeted treatment strategies.

## AIM OF REVIEW

To discuss the role and “state of the art” utility of pharmacodynamic biomarkers in early phase clinical trials with special emphasis on the tissue-based approaches and technology platforms used with specific examples of their use in clinical trials of novel targeted agents.

## Figures and Tables

**Fig. (1):**
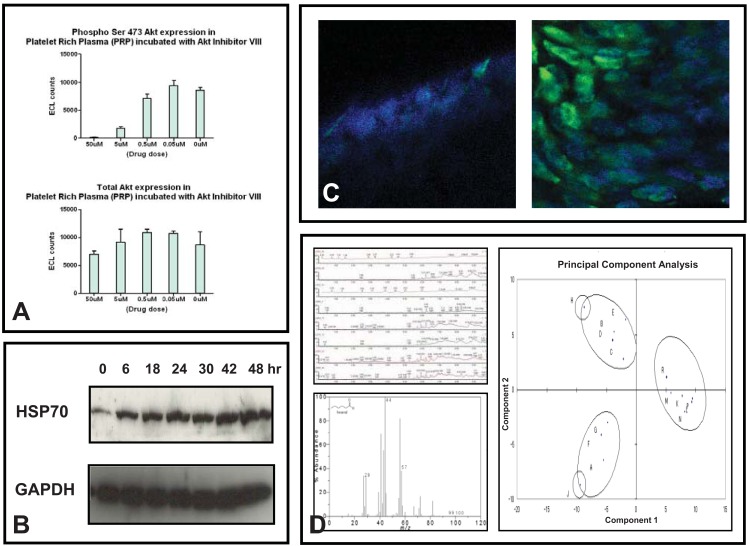
**A**) Changes in phosphorylated-Akt levels in platelet rich plasma from samples incubated with an Akt inhibitor ex vivo, studied using ELISA. **B**) Demonstration of the increase in HSP70 protein levels in PBMCs after treatment with 17-AAG by western blotting. **C**) Hyperacetylation in plucked hair follicles (pre- and post-treatment in the left and right panels, respectively) after treatment with a HDAC inhibitor demonstrated by immunofluorescence. **D**) Relevant readouts of liquid chromatography-mass spectrometry profiles and the software used in data analysis.

**Fig. (2):**
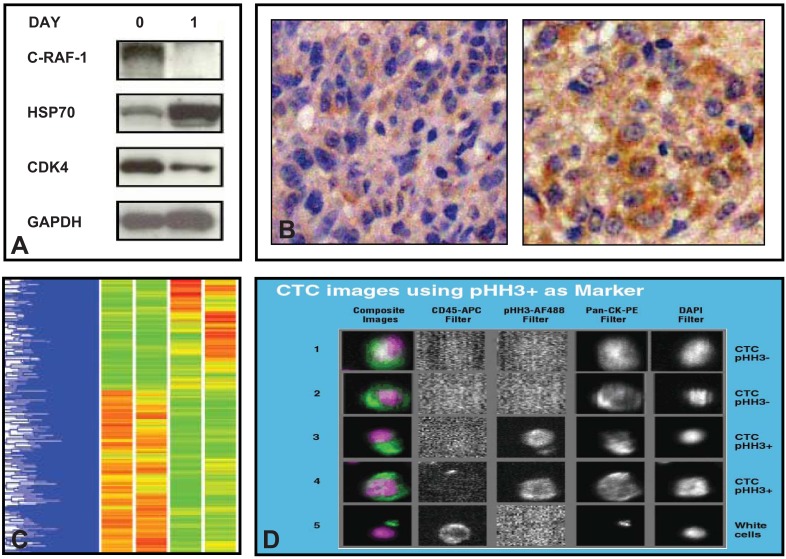
**A**) Western blotting demonstrating changes in client proteins and co-chaperones of HSP90 in pretreatment and post treatment samples of patients treated with an HSP90 inhibitor, 17-AAG. **B**) Use of immunohistochemistry to study an increase in protein levels of HSP70 in pre and post treatment samples of patients treated with a HSP90 inhibitor 17-AAG. **C**) Use of c-DNA microarray to study mRNA array expression signatures in pre and post treatment samples of patients treated with a HSP90 inhibitor 17-AAG. **D**) Use of CTCs in the study of phosphohistone-H3 (pHH3), a marker of mitotic arrest (Courtesy of Dr. David Olmos, The Institute of Cancer Research, UK).
